# Long range chromosome organization in *Escherichia coli*: The position of the replication origin defines the non-structured regions and the Right and Left macrodomains

**DOI:** 10.1371/journal.pgen.1006758

**Published:** 2017-05-09

**Authors:** Stéphane Duigou, Frédéric Boccard

**Affiliations:** Institute for Integrative Biology of the Cell (I2BC), CEA, CNRS, Univ. Paris‐Sud, Université Paris‐Saclay, Gif‐sur‐Yvette, France; Universidad de Sevilla, SPAIN

## Abstract

The *Escherichia coli* chromosome is organized into four macrodomains (Ori, Ter, Right and Left) and two non-structured regions. This organization influences the segregation of sister chromatids, the mobility of chromosomal DNA, and the cellular localization of the chromosome. The organization of the Ter and Ori macrodomains relies on two specific systems, MatP/*matS* for the Ter domain and MaoP/*maoS* for the Ori domain, respectively. Here by constructing strains with chromosome rearrangements to reshuffle the distribution of chromosomal segments, we reveal that the difference between the non-structured regions and the Right and Left lateral macrodomains relies on their position on the chromosome. A change in the genetic location of *oriC* generated either by an inversion within the Ori macrodomain or by the insertion of a second *oriC* modifies the position of Right and Left macrodomains, as the chromosome region the closest to *oriC* are always non-structured while the regions further away behave as macrodomain regardless of their DNA sequence. Using fluorescent microscopy we estimated that loci belonging to a non-structured region are significantly closer to the Ori MD than loci belonging to a lateral MD. Altogether, our results suggest that the origin of replication plays a prominent role in chromosome organization in *E*. *coli*, as it determines structuring and localization of macrodomains in growing cell.

## Author summary

Chromosomes allow the genetic information to be stored, transmitted and organized inside the cell. In bacteria, chromosomes are generally circular and they are shaped and organized by several mechanisms allowing simultaneous transcription, replication and segregation. The way such fundamental processes are managed is still unclear, but in the Gram negative bacteria *Escherichia coli*, one level of chromosome organization relies on a large scale structuring of the chromosome in macrodomains and non-structured regions. Macrodomains have been defined as chromosomal domains of megabase (Mb) sized genetically isolated from each other. *E*. *coli* chromosome is divided in 4 macrodomains (Ter, Ori, Right and Left) and two right/left non-structured regions (NSR, and NSL). Factors that organize Ter and Ori MD have been identified and characterized previously: MatP structures the Ter MD by binding to 23 *matS* sequences disseminated in the Ter MD, and MaoP together with the unique sequence *maoS* organises the Ori MD by an unknown mechanism. In constrast, we show here by reshuffling the chromosome that the Right and Left MD as well as the NSR and NSL regions are defined by their chromosomal location and that the chromosomal position of *oriC* defines the position and the extent of the NS regions and Right/Left macrodomains.

## Introduction

Genetic information is stored in long DNA polymers that need to be organized and strongly compacted to form functional chromosomes. In bacteria, chromosomes are generally circular and range from 0.4 to 8 Mb in length. General mechanisms at work for the folding and compacting of bacterial chromosomes remain elusive even if several layers of organization have been identified from the plectonemic structure generated by negative DNA supercoiling to large chromosomal domains [1,2]. At the small scale, abundant nucleoid associated proteins (NAPs) bind DNA and assist chromosome condensation. In *E*. *coli*, major NAPs are H-NS, HU, Fis, IHF, and StpA. By bending, wrapping or bridging DNA, they play a major role not only in DNA compaction but also in regulation of transcription, and replication [3,4]. At larger scale, recent advances in chromosome conformation capture methods have enable high resolution studies of chromosome structure *in vivo* and revealed in bacteria the presence of domains averaging 100 kilobases (kb), called “Chromosomal Interaction Domains” or CIDs [5]. Within one CID, loci interact more frequently with each other than with loci in other CIDs. In *Caulobacter crescentus*, 23 CIDs have been identified, ranging in length from 30 to 420 kb [6]. These CIDs are also found, with a comparable frequency, in *Vibrio cholerae* (20 CIDs in chrI, 7 CIDs in chrII [7]), and in *Bacillus subtilis* (20 CIDs in [8] and 25 CIDs in [9]). In *B*. *subtilis* and *C*. *crescentus* these domains appear to be nested within larger domains of megabases length [9,10]. Indeed, a 3D topological model of the *B*. *subtilis* chromosome reveals the presence of three large domains: (i) the origin domain, a 1.4 Mb domain that encompasses the origin of replication; (ii) on both sides the middle region where the two arms of the chromosome are in very close proximity; and (iii) a smaller domain of 500 kb containing the terminus of replication. The molecular bases of this two scales of domains (CID and large domain) remain elusive. Authors propose that CIDs are delimited by plectonemic free regions [10] or by long highly transcribed genes [11], and the Ori domain of *B*. *subtilis* may participate to the control of replication initiation [9].

**Fig 1:**
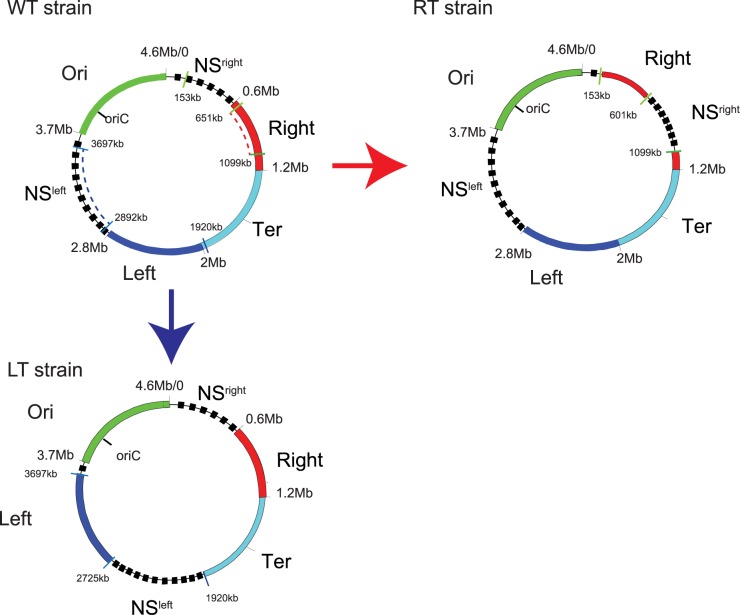
Chromosomal rearrangement by transposition. Chromosomal map of the 4 MDs (Ori: green, Right: red, Ter: light blue, Left: Dark blue) and the two non-structured regions (dashed black line) in the WT strain, the Right transposed strain and the Left transposed strain. *att* sequences used for the RT transposition are indicated by the green barre, the Right fragment between *attR127* (651 kb) and *attLlc13* (1099 kb)(dashed red line) was transposed at the position *attB O-NSR* (153 kb). *att* sequences used for the LT transposition are marked in blue, the NSL fragment between *attR124* (2892 kb) and *attL146* (3697 kb, dashed blue line) was transposed at the position *attB LT2* (1920 kb).

In *E*. *coli*, 24 CIDs have been identified [12] and as in *B*. *subtilis*, these CIDs are nested in larger domains of Mb-sized previously identified. Ori and Ter domains were first identified by characterizing subcellular positioning of 23 chromosomal loci in *E*. *coli* [13]. The Ter domain is centered on *dif*, a 28bp sequence allowing chromosome dimer resolution and the Ori domain asymmetrically surround *oriC*. This domain map was completed by a genetic approach based on λ phage site specific recombination, which reported the frequency of collisions between different chromosomal loci [2]. This approach allowed the identification of yet another level of organization with the definition of macrodomains (MDs) as regions genetically isolated from other MDs, where loci interact more frequently with loci from the same MD than with other MD. Four MDs were identified, two of which coincide with the Ori and Ter regions identified by Niki and *col*. Thus, each chromosome arm between Ori and Ter MD is divided in two regions, a non-structured (NS) region and a MD (Fig 1). The two NS regions called NS Right (NSR) and NS Left (NSL) are adjacent to the Ori MD and the two lateral MDs called Right and Left are adjacent to the Ter MD (Fig 1). MDs and NS regions exhibit different features: (i) NSR and NSL interact with Ori MD and their adjacent lateral MD [2], (ii) loci composing the NSR and NSL regions present an apparent mobility higher than loci composing MD [14], (iii) following replication, duplicated loci belonging to NS regions segregate in a short period of time and concomitantly with the Ori MD [14]. Inversely (i) MDs interact only with their adjacent NS regions but not with other MDs, (ii) the mobility of loci composing MD appear constrained, (iii) following replication, duplicated loci belonging to MDs show a longer colocalization step.

The molecular mechanisms responsible for this organization in MDs have been partially uncovered. The structuring of the Ter MD relies on the binding of MatP to 23 *matS* sequences distributed all along the Ter domain [15]. Localization and segregation of this MD are tightly controlled during the cell cycle: the Ter MD is anchored to the division machinery through an interaction of MatP with ZapB [16], a component of the divisome, and the DNA translocase FtsK removes MatP from the Ter MD to achieve chromosome segregation [17]. In the absence of MatP, the Ter MD interacts with the adjacent Right and Left MDs, loci belonging to the Ter exhibit a higher level of mobility, and segregation of the Ter MD occurs earlier in the cell cycle [15,18]. The organization of the Ori MD depends on the MaoP protein and on a single sequence of 17 bp, called *maoS*, located close to *oriC*. How the *maoS*/MaoP system structures the DNA over several hundred of kilobases is unknown. Nevertheless, in a *ΔmaoP* strain, the Ori MD interacts with the Right MD, loci belonging to this MD exhibit a higher level of mobility, and the segregation timing of the Ori MD is affected [19].

To understand the molecular events that underlie the existence of NS regions and the two lateral MDs on the right and left replichores, we engineered different chromosome configurations that alter the localization of NS regions and MDs on the chromosome. We show that the structuring of Right and Left MD depends on their position on the chromosome suggesting that no specific DNA sequence determinants contained into these MDs is required. We demonstrate that displacement of *oriC* by inversion of the Ori MD or by introduction of a second replication origin changes the interaction pattern over a long distance and redefines the limits of the lateral MD and the NS regions. Finally, we also show that displacement of *oriC* leads to the repositioning of chromosomal loci inside the cell, bringing in a closer proximity regions that can genetically interact with the Ori MD.

## Results

### Structuring of the Right MD relies on its position on the chromosome

To understand if protein binding sites organize the Right and Left MDs we used a statistical approach that was previously successfully used to allow the identification of the *matS* sequences disseminated along the Ter MD [15]. It consisted of evaluating the exceptionality of DNA motifs between 8 to 15 bp in the Right and Left MD compared to their exceptionality outside these MDs. We were unable to identify such specific determinants suggesting that the structuring of Right and Left MDs may rely on a mechanism independent of their sequence. If Right and Left MD organization does not depend on the recognition of a MD-specific motif, a likely hypothesis is that the chromosomal position of these lateral MDs may isolate these domains from the others MD. To test this hypothesis, we used a method called “transposition” as it mimics conservative transposition events by relocating large chromosome segments at specific positions on the genetic map (see Materials and Methods section and [20]). As described before, such rearrangements allow the preservation of gene orientation and have no dramatic effect on cell physiology [20]. We then determined, in different chromosomal configurations, the interaction pattern of chromosomal loci belonging to these transposed segments by monitoring the recombination efficiency between several *att*R/L sites derived from the λ site specific integration module.

**Fig 2:**
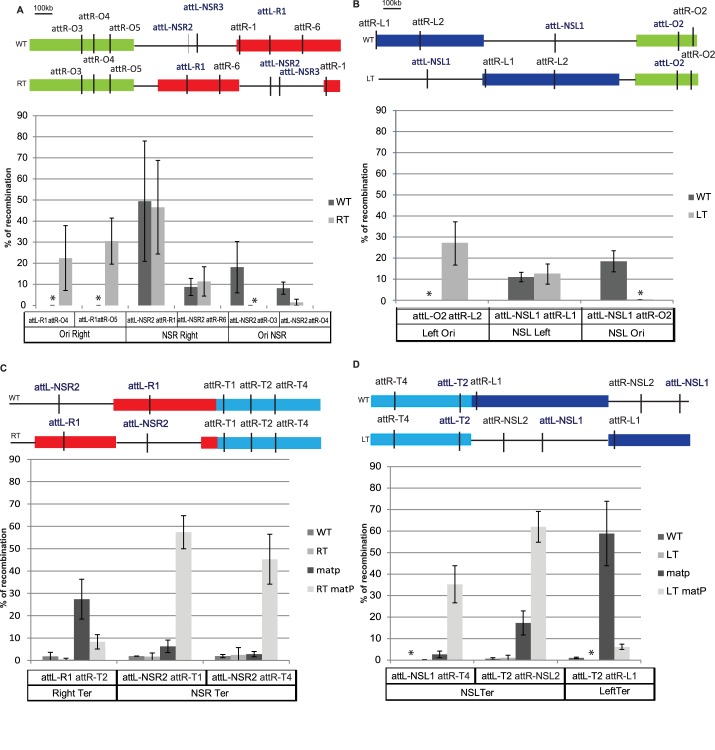
Effect of Right and Left transposition on long distance DNA interactions. Histograms of recombination frequency between *attL* and *attR* sequences in WT, RT, and LT strains. The y-axis indicates the percentage of recombination between *attL* and *attR* sequences, obtained as described in Materials and Methods with 20’ induction at 36°C. The relative position of each *att* sequence used in the experiment is represented on the MD map on top of each panel. Histograms show the average of at least 3 independent experiments with their respective standard-deviation. Frequencies obtained in the WT strain are represented with the black bars, in the RT strain (A,C) or LT strain (B,D) with the gray bars and in the Δ*matP* RT strain (C), or Δ*matP* LT strain (D) with the light gray bars. * indicates that no *att*L*-att*R recombinant was obtained.

First, we used an *E*. *coli* strain in which most of the Right MD (651 kb-1099 kb) is transposed at position 153 kb (Right-Transposed (RT) strain; Fig 1) [20]. This transposition places most of the Right MD in close proximity to the Ori MD, and most of the NSR close to the Ter MD. We tested in this RT strain if chromosomal loci from the Right MD retain a low probability to interact with chromosomal loci from the Ori MD, by measuring the recombination rate between *att*R/L sequences inserted in Ori and Right MDs. As a control, we also tested interaction properties of chromosomal loci from the NSR region. In wild-type (WT), the *attR/L* sequences inserted in the Right and Ori MDs are able to recombine with a *attR/L* inserted in the NSR region, but not with each other (Fig 2A and [2]). In contrast, in the RT strain, an *attL* sequence inserted in the Right MD (*att*L-R1) recombines with a high efficiency with *attR* sequences located in the Ori MD (*att*R-O4/*att*RO-5), but an *att*L sequence located in the NSR region (*att*L*-*NSR2/ *att*L*-*NSR3) does not recombine with *att*R sequences in the Ori MD (*att*R-O4/*att*R-O3). Frequencies of interactions between *att*R/L sequences in the NSR region and the Right MD are similar to the WT strain. These results show that the interactions between Right and Ori MD become more frequent when the Right MD is relocated near the Ori MD, and that interactions between NSR and Ori MDs are lost when NSR is relocated far from the Ori MD. These results suggest that the MD and NS properties of the Right MD and NSR region relies on their position on the chromosome and not on specific genetic determinants.

### Structuring of the Left MD also depends on its chromosomal position

We used a similar approach to test whether the structuring of the Left MD also depends on its localization on the chromosome. We rearranged the chromosome by transposing the NSL region (from 2.892 kb to 3.697 kb) between the Ter and Left MDs, at position 1.920 kb (Fig 1). In this strain, called LT for Left Transposed, the Left MD is located between the Ori MD and the NSL region, and the NSL region flanks the Ter MD. We tested the ability of chromosomal loci located in Left MD and in the NSL region to interact with chromosomal loci located in the Ori MD (Fig 2B). In a WT strain, *attR/L* sequences inserted in the Left and Ori MDs are able to recombine with *attR/L* sequences inserted in the NSL region, but not with each other (Fig 2B and [2]). In contrast, in the LT strain, *attL/R* sequences located in the Ori MD (*attL-O2*, *attR-O2*) recombined efficiently with a complementary *att* sequence located in the Left MD (*attR-L2)* but not with one in the NSL region (*attL-NSL1)*. This indicates that in LT strain chromosomal loci from the Left and Ori MDs are now able to interact with each other whereas loci from the NSL region do not collide with loci from the Ori MD. These results show that, as observed for the Right MD, interaction properties of the Left MD and of the NSL region rely on their position on the chromosome.

### Long distance DNA interactions are restricted by MatP in the Ter MD but not in the Right MD

We also tested in these transposed strains the ability of non-structured regions and MDs to interact with the Ter MD. In the WT strain, the Ter MD is isolated from the neighboring Right and Left MD and from the NSR/NSL regions. This insulation relies only on the Ter structuring and on the action of MatP (Fig 2C and 2D, [15]). In the LT and RT strains, chromosomal loci in the transposed NS regions (attL-NSR2 in NSR and *att*R-NSL2/*att*L-NSL1 in NSL) do not interact with chromosomal loci from the Ter MD (*att*R-T1, *att*R-T4 and *att*L-T2) (Fig 2C and 2D). Nevertheless, deletion of Δ*matP* allows NSR/Ter, NSL/Ter interactions, in these strains.

Altogether these results show that NSR and NSL regions repositioned close to the Ter MD behave as MDs and conversely Right and Left MD in RT and LT strain behave as NS regions. This indicates that the structuring of the lateral MDs and NS regions relies on their location on the chromosome and that the region adjacent to the Ori MD is non-structured and the region adjacent to the Ter MD is structured regardless of their sequence composition. The phenomenon triggering the structuring of sequences in Right MD might be a long distance effect coming either from the Ori MD or from the Ter MD. The Ter MD is associated with the division machinery during a part of the cell cycle through an interaction between the component of the divisome ZapB and MatP [16]. This anchorage has an impact on the dynamic of the DNA several hundreds of Kb away [20]. To test if the structuring of the Right MD relies on an effect spreading from the Ter MD, we tested the ability of chromosomal loci from Right and Ori MDs to interact with each other in a Δ*matP* mutant. In the WT strain, chromosomal loci from the Right MD are not able to interact with chromosomal loci from the Ori MD as shown in S1 Fig: an attR-R4 sequence inserted in the middle of the Right MD interacts with an *attL* sequence located in the NSR region (*att*L-NSR1), but interact poorly with an *att*L sequence located at the boundary of the Ori MD (*att*L-O7) or not with a attL sequence in the Ori MD (*att*L-O6). In the Δ*matP* mutant, chromosomal loci from Right and Ori MDs behave as in the WT strain and *attR-NSR4* recombines efficiently with an *attL* sequence located in the NSR region (*att*L-NSR1), but not with *att*L sequences located in the Ori MD (*att*L-O6 *and att*L-O7). This result shows that the interaction pattern of the Right MD with the Ori MD is independent of MatP and of the structuring of the Ter MD.

### The NSR-Right transitions occurs at a constant distance from o*riC*

**Fig 3:**
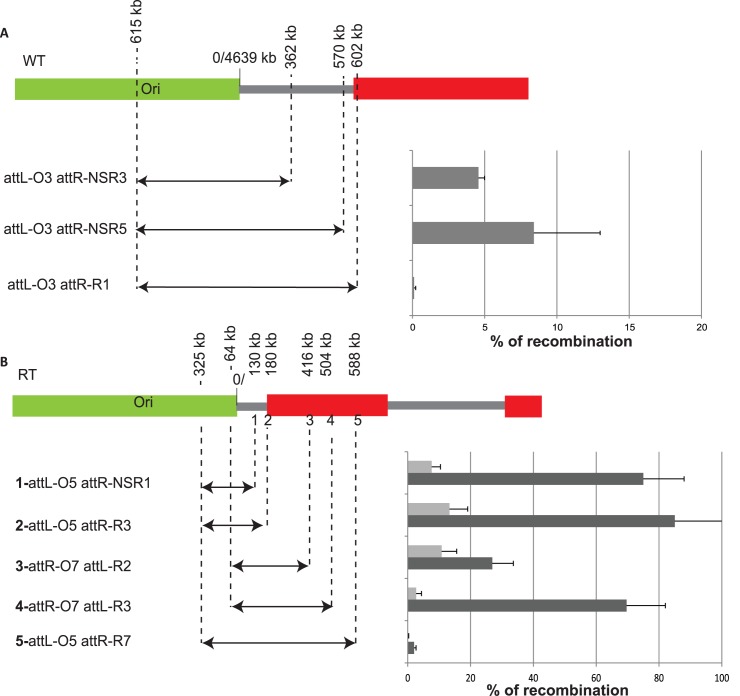
The interaction limit of the Ori MD. Graphical representation of the Ori MD (green box), NSR region (gray line) and Right MD (red box). Coordinates of the *attR/L* sequences are indicated in fonction of their distance from the zero pb reference of the MG1655 genome for both configuration WT and RT. Percentages of recombination between *attL* and *attR* obtained after induction of 20’ at 36°C or 10’ at 37°C are shown. The histograms correspond to an average of at least 3 independent experiments with standard-deviations.

The structuring of lateral domains and NS regions rely on their chromosomal position but are independent of the Ter MD and of the MatP/*matS* system. To better understand the nature of this structuring, we undertook the accurate identification of the boundaries between the Right MD and the NSR region in the RT strain. On the right replication arm, the NSR/Right MD boundary is defined by the interaction limit of the Ori MD. In a WT background, the last interacting sequence with the Ori MD (*attR-NSR5*, 570 kb) and the first non-interacting sequence *(att*R-R1 602 kb) (Fig 3A, [2]) define a 32 kb interval as the limit of interactions. To define the boundaries between the Right MD and the NSR region in the RT strain, we used 5 different *att*L/R sequences inserted between positions 130 kb to 588 kb (names 1 to 5, Fig 3B) and measured their ability to recombine with two *att*L/R sequences inserted in the Ori MD (*att*L-O5, *att*R-O7). Results suggest that the interaction limit of the Ori MD is defined by *att*L-R3 (n°4) and *att*R-R7 (n°5) (Fig 3B). This result was confirmed by the use of a higher level of recombinase which increased the percentage of recombination, as previously described [2]. The recombination rates are presented in Fig 3B and confirm that the interaction limit of the Ori MD is located between 504 and 588 kb. Remarkably, this limit is comprised inside the sequence of the transposed Right MD confirming that the structuring of the Right MD does not rely on a sequence determinant. Moreover, in the WT and RT strains, the interaction limit with the Ori MD is located at a similar distance (602 kb in wt, 588 kb in RT) from the right border of the Ori MD suggesting that the structuring of the Right MD might be a long distance effect originating from the Ori MD. Structuring of the Left MD, could also rely on the same mechanism.

### Displacement of *oriC* changes the pattern of genetic interactions of the Ori MD

**Fig 4:**
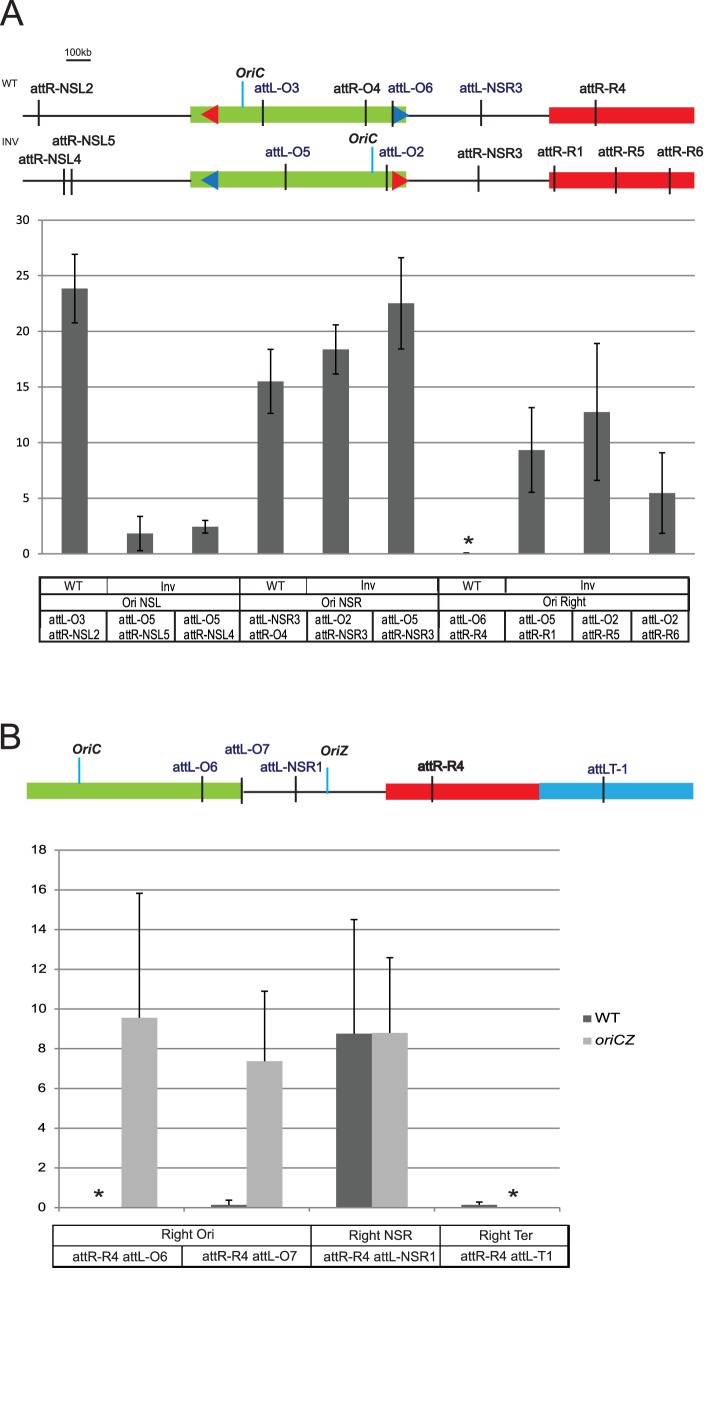
The position of the replication origin changes the Right and Left boundaries. Histograms of recombination frequencies between *att*L and *att*R sequences in WT, and Inv strain. Recombination frequencies between different *attR/L* sites are indicated on the y-axis. These values are the average of at least 3 independent experiments and the error bars correspond to the standard deviation. For both panels, strains were grown in minimal medium and the recombinase production was obtained by shifting cultures at 38°C for 10’. Relative position of each *att* sequence used in the experiment is represented on the MD map on top of each panel. The name of the *att* sequence, and the MD which they belong to, are indicated on the x-axis. The genetic backgrounds are indicated below the histogram (A), or with a color code (B). * indicates that the recombination frequency obtained was repeatedly 0.

Right and Left MDs are delimited on one side by the Ter MD and on the other side by the interaction limit with the Ori MD. Since NSR and NSL region are able to interact with the flanking MD, chromosomal loci from Ori MD can interact with loci localized between the Left/NSL limit (2.8 Mb) to the NSR/Right limit (600 kb) (Fig 1). Strikingly, this interacting zone of 2.4 Mb is approximately centered on *oriC* (3.92Mb), in contrast to the Ori MD itself. To test if the distance to *oriC* is important, we displaced *oriC* close to the NSR region by inverting most of the Ori MD between positions 3.85 Mb and 4.64 Mb, giving rise to the *Inv* strain [21]. In this chromosome configuration, *oriC* is placed at 4.57 Mb instead of 3.92 Mb on the genetic map. This strain exhibits a slight growth defect in rich medium linked to an unbalance of replication arm, but no apparent phenotype in synthetic Medium (SM) [21]. We measured in these growth conditions the frequency of interactions between the Ori MD and *att*L/R sequences inserted along the right replication arm. Strikingly, in the Inv strain, *att*L-O2 or *att*L-O5 inserted in the Ori MD interact readily with *attR* sites located either in the NSR region (*attR-NSR3 -*362kb) or in the Right sequence up to the distance of 1 Mb (*att*R-R1 602 kb, *att*R-R5 884 kb, and *att*R-R6 1000 kb) (Fig 4). Thus, after displacement of *oriC*, *attL* sequences inserted at different positions in the MD Ori are able to interact with *att*R sequences located in both the NSR region and the Right MD. These results show that displacing the origin of replication closer to the NSR region extends the interaction range of the Ori MD on the right replication arm.

Moreover, in the *Inv* strain, *oriC* is located further away from NSL region. Therefore, if the distance from *oriC* is responsible for defining the interaction limit of the Ori MD, the limit between NSL and Left MD should be closer to the Ori MD in the *Inv* strain. We tested this hypothesis by measuring the recombination frequency between *att* sequences located in the Ori MD and the NSL region. Results shown in Fig 4A demonstrate that in the *Inv* strain, *att*L sequences inserted in the Ori MD (*att*L-O2, *att*L-O5) recombine hardly (10 times less than in a WT strain) with *att*R sequences located in the NSL region (*att*R-NSL4 at 3.25 Mb and *att*R-NSL5 at 3.28 Mb) indicating a change in the position of the interaction limit in the NSL region. Altogether these results suggest that the genetic distance from *oriC* defines an “interaction zone” in which loci can interact with the Ori MD, and that this interaction zone delimits the Right and Left MDs and the NSR / NSL regions.

### The *oriC* position defines the extent of the Right MD

Inverting most of the Ori MD does not only displace *oriC* but also modifies the position of all the Ori MD loci. To test whether the displacement of *oriC* is sufficient to affect the structuring of the Right MD, we used a strain carrying a second origin of replication [22] and P1 transduced this origin into our strain. This strain, called *oriCZ*, exhibit a replication profile where both origins fire equally [23], and a slight growth defect in rich medium but can grow without major defects in SM [24].

We measured in SM the interaction frequencies of loci located along the right arm of the chromosome (Fig 4B). In the *oriCZ* strain, *att*R-R4 (806 kb) located in the Right MD recombines with a high efficiency with *att*L-O6 (4501 kb) and *att*L-O7 (33 kb) located in the Ori MD (Fig 4B). These results show that loci located in the Ori and Right MDs, which cannot interact in the WT strain, are able to interact in the presence of an additional replication origin located nearby. In the same strain, *att*R-R4 and *att*L-T1 (1461 kb) are not able to interact, showing that genetic isolation of the Ter MD is not affected, and that the proximity with a replication origin cannot counteract the action of MatP. At this position, *oriC* and the neighboring 5 kb present in the *oriZ* cassette enable the collision between sequences of the Ori and Right MDs. Remarkably these results demonstrate that the proximity of the origin of replication itself confers a “Non Structured region” behavior to the sequences of the Right MD.

### Ori MD is located closer to the NSR region than to the Right MD

**Fig 5:**
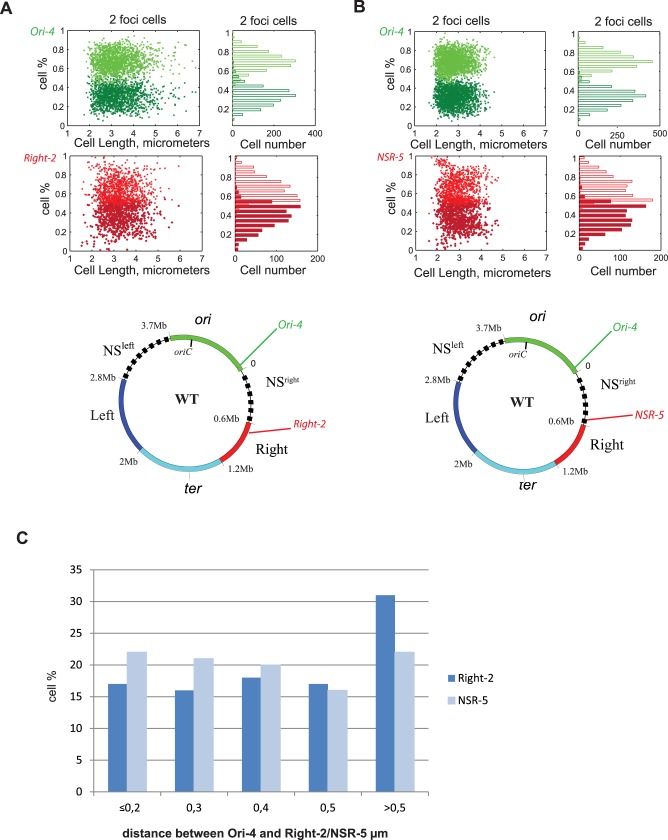
Position of chromosomal loci in the Ori, NSR region and Right MD. MD maps of the *E*. *coli* chromosome are represented with the position of the Ori-4, Right-2, NSR-5 FROS-Tag in a WT strain. For each panel, the position of foci in cell containing 2 foci, are represented in function of the long axis of the cell (y-axis) and in function of the cell length (x-axis). Green dots and bars show the position of the Ori-4 loci and red dots and bars correspond to the localization of the Right-2 (A) or NSR-5(B) loci. (C) Histograms of the percentage of the population presenting different interfocal distance (<0.2 to > 0.5) between Ori-4 and Right-2 (dark blue) or Ori-4 and NSR-5 (light blue).

Considering that the recombination system that we use to measure interaction frequency rely on spatial proximity between *att*L and *att*R sites, we decided to assess the position of different loci inside the cell by a complementary approach. We used FROS tags (*parS*^*P1*^ and *parS*^*T1*^) to simultaneously observe loci located in the Ori MD (Ori-4 at 9.9 kb [14]) and loci located in the NSR region (NSR-5 at 515 kb [14]) or in the Right MD (Right-2 at 738 kb [14]) and analyzed their position inside growing cells. In the WT strain, Ori-4 loci are distributed in two peaks around the positions 0.35/0.7 relative to long axis of the cell (Fig 5A and 5B). Right-2 and NSR-5 share a common distribution pattern and foci are distributed in two broad peaks between the position 0.4/0.65 relative to the cell length (Fig 5A, S2A Fig, S3A Fig). Strikingly Right-2 and NSR-5 exhibits almost no differences of cellular localization even if they exhibit strong differences of interaction with the Ori MD. We measured the distance between these two loci and Ori-4 (Fig 5C), and noticed that NSR-5 is localized significantly closer to Ori-4 than Right-2. Indeed, the inter-focal distance measured between Ori-4 and NSR-5 and Ori-4 and Right-2 show that the percentages of cells where the two foci are in close proximity (bellow 0.3 μm) is more important for Ori-4/ NSR-5 (43%) than for Ori-4/Right-2 (33%) and foci Ori-4/NSR-5 are co-localized (interfocal distance below 0.2μm) in 22% of the cell population when Ori-4/Right-2 co-localized in 17% of the cells. Inversely the percentage of cells where the two foci are far away (>0.5 μm) is more important for Ori-4/Right-2 (31%) than for Ori-4/ NSR-5 (22%). Furthermore statistical analysis of the distributions of interfocal distances in the population with a Kolmogorov-Smirnov test (p = 0.005) allowed us to conclude that Ori-4 is significantly closer to NSR-5 than Right-2. These results suggest a correlation between the ability of loci to genetically interact and their physical proximity inside the cell.

### Displacing *oriC* leads to repositioning of chromosomal loci inside the cell

**Fig 6:**
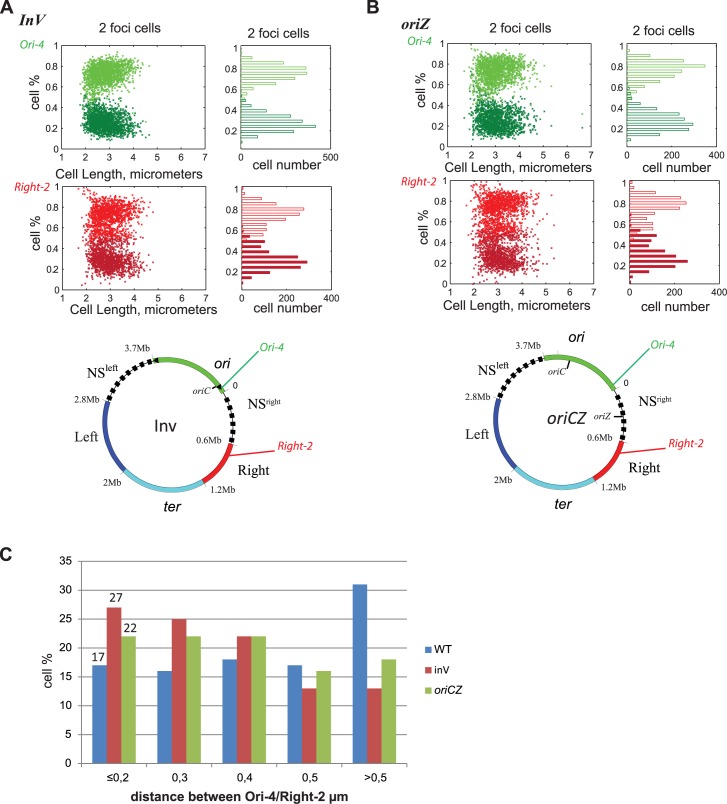
Position of chromosomal loci in the Ori and Right MD depending of *oriC* position. MD map of the *E*. *coli* chromosome are represented with the position of the Ori-4, Right-2, FROS-Tag in an Inv (A), or *oriCZ* (B) configurations. For each panel, the position of foci in cell containing 2 foci (cell with one focus are show in S4 Fig), are represent in function of the long axis of the cell (y-axis) and in function of the cell length (x-axis). Red dots and bars correspond to the localization of the Right-2 loci and green ones to the Ori-4 loci. (C) Histogram of the percentage of the population presenting different interfocal distance (<0.2 to > 0.5) between Ori-4 and Right-2 in the WT strain (blue), Inv strain (red) and *oriCZ* strain (green). Numbers on the first columns show the percentage of cells where both foci are co-localized.

The position of the origin of replication at 4.57 Mb in the *Inv* strain or at 344 kb in the *oriCZ* strain allows interactions between sequences present in Ori and Right MDs. A possible explanation could be that the proximity of the replication origin triggers a repositioning of the sequences of the Right MD in closer proximity with the Ori MD. To test this hypothesis, we analyzed the cellular position of Ori-4 and Right-2 in the two strains Inv and *oriCZ*. In the Inv strain the cellular position of Ori-4 foci is slightly modified by *oriC* proximity, and exhibits a more polar localization (Fig 6A, S2 Fig). Indeed the two peaks of Ori-4 loci are positioned around the 0.25/0.8 positions relative cell length. The localization of Right-2 foci is drastically modified and, instead of being broadly distributed in the middle of the cell as in the WT strain, Right-2 loci are localized in two peaks at the 0.3/0.8 position relative to the cell length. These results show that inversion of the Ori MD has a mild effect on the localization of Ori MD loci and a strong effect on Right MD loci. The introduction of a second origin of replication, *oriZ*, triggers a similar repositioning of chromosomal loci from Ori and Right MDs (Fig 6B, S3 and S4 Figs)). Indeed, in the *oriCZ* strain, Ori-4 foci are localized around the 0.25/0.8 position relative to the cell length, and Right-2 foci are distributed in two peaks around the 0.3/0.8 position relative to the cell length. In both Inv and *oriCZ* strains, Right-2 distribution further overlaps with Ori-4 as the inter-focal distance between these foci decreases significantly compared to the WT (Fig 6C).

These results show that in both Inv and *oriCZ* genetic backgrounds, where the origin of replication is closer to the Right MD on the chromosome, Right loci are re-localized closer to the Ori MD in growing cells. Therefore we observe a strong correlation between the cellular position of Right and Ori MD and their ability to interact genetically.

## Discussion

This work provides insights into the biological processes controlling *E*. *coli* chromosome organization in MDs and unstructured regions. We have shown that this chromosome organization involves two different kinds of MDs: on the one hand, Ter and Ori MDs for which the structuring relies on specific factors such as MatP/*matS* and MaoP/*maoS* respectively, and on the other hand Right and Left MDs for which the structuring depends on their position on the chromosome. We showed that DNA regions between the Ori and Ter MDs are defined as lateral MDs or non-structured regions exclusively in regard of their genetic distance from the origin of replication: proximal sequences behave as NS regions and distal sequences as MDs. This work sheds light on the mechanism structuring the lateral MDs and the NS regions, allowing us to have a more complete view of the mechanisms structuring the chromosome in MDs.

The Ter MD is the most isolated MD, and does not interact with any other chromosomal region, neither with the adjacent lateral MD, nor with the NS regions [2,12]. This strong isolation is associated with a specific level of condensation [12,18], a cellular anchorage to the divisome machinery triggering a polar localization during a long period of the cell cycle [16], and a late segregation timing just before division [14]. All these properties rely on the action of MatP, structuring the Ter by binding over the 23 *matS* sequence. Here we show that even the relative proximity of a second origin of replication (*oriZ*) does not allow interactions of Ter loci with non-Ter loci.

At the opposite side of the chromosome, the Ori MD is organized by MaoP and the 17 bp *maoS* sequence. In a Δ*maoP* strain, the Ori region loses this MD property[19]. In this genetic background, long range interaction between Ori and Right MD are detected at a high level although the genetic distance between Right and *oriC* is unchanged. Nevertheless, *maoS* is not able to extend the range of interactions of the Ori MD because the introduction of a second *maoS* sequence in the NSR does not allow interactions between Right and Ori MDs (S5 Fig). Inactivation of MaoP/*maoS* affects as well the timing, positioning and separation of markers in the Ori MD, maintaining Ori loci together and in the middle of the cell and delaying the segregation to the 0.27/0.75 positions relative to cell length. In contrast, inactivation of MaoP had no effect on the position of Right marker [19]. Altogether these suggest that in Δ*maoP* background, Right and Ori MDs share a closer proximity and a common localization over a longer period of the cell cycle. This proximity could explain the higher level of interactions between Right and Ori MDs, as we observed the same correlation of cellular localization and interactions in Inv or *oriCZ* backgrounds.

The Right and Left MDs are defined by their incapacity to interact with the other MDs. Since the Ter MD is isolated from any other domain, the definition of the Right and Left MDs rely only on the range of interaction of the Ori MD. This range of interactions has been characterized on the right arm of the chromosome with strains harboring different chromosomal configurations (WT and RT) and corresponds to a region of few tens of kb separating Right and NSR. This region is, in both contexts, at a similar position around 600 kb, and at 1.3 Mb of *oriC*. On the left arm of the chromosome, the first non-interacting point with the Ori MD is also at 1.3Mb of *oriC* at the position 2.61 Mb [2]. Altogether these results show that an “interacting zone” encompassing half of the chromosome is defined by the distance to the origin of replication. This zone is composed, in a WT strain, of the Ori MD and of the two Non-structured regions NSR and NSL and is centered on *oriC*. The “interacting zone” follows *oriC* as observed in the *Inv* strain where this zone is displaced on the right chromosome arm as *oriC*, allowing interactions between Ori and Right MD and restricting interactions between NSL and Ori MD. Adding a second origin of replication, as in the *oriCZ* strain, extends this zone through the Right MD up to the Ter MD. Lateral MD can be defined as the sequences that are outside of the Ter MD and outside of the Ori MD interacting zone. Conversely NS regions are in the Ori MD interacting zone but outside of the Ori MD and they are not affected by MaoP.

How the origin of replication impacts the chromosome structuring over a distance of several hundred of kilobases is unknown, but may involve among other hypotheses, a mechanism of cellular localization of the sequences surrounding *oriC*. This hypothesis is sustained by the fact that loci from the Right MD are re-localized in closer physical proximity to loci from the Ori MD in the *oriCZ* or Inv strains, two strains in which chromosomal loci from Ori and Right MDs can interact. This result shows that the position of the origin of replication on the genetic map has a direct impact on the cellular localization of Right MD and NSR region (Fig 6, S3 Fig, S4 Fig). The “interacting zone” defined by the genetic interaction could also, under this hypothesis, be assimilated as a cellular territory.

Replication and segregation timing of chromosomal loci are also directly affected by the position of the origin of replication. In a WT strain, the segregation of the Ori MD is delayed after replication and NS regions and Ori MD segregate together [14,25,26]. This concomitant segregation could also be a factor promoting the interactions between these two regions. In Inv and *oriCZ* strain, the replication/segregation timing of the Right MD happens earlier in the cell cycle as the number of cells with 2 Right foci increases compare to WT. This earlier segregation may drive a concomitant re-localization of Right and Ori loci that could also stimulate the interaction between these MDs.

## Materials and methods

### Strains and media

The bacterial strains used in this study are listed in S1 Table. *E*. *coli*. As previously described in [19]” strains were grown at 30°C in Lennox broth (LB), or in synthetic medium A supplemented with 0.12% of casaminoacids and 0.2% of glucose (SM). Antibiotics were added when necessary. Transposed strains were constructed as described in [20], the *att*B, *att*L’, *att*R’ sequence were successively replaced by a rifampicin resistant gene between two *frt* sequences using the deletion- insertion one-step technique in strain carrying the plasmid pKD46 [27] followed by expression of FLP recombinase with pcp20 to delete the antibiotic resistance gene [28]. Deletion/insertion coordinates are indicated in S1 Table. Constructions of strains were verified by PCR.

### Inversion assays

Strains were transformed with pTSA29-CXI [2] a plasmid expressing lambda *int* and *xis* under the control of *cI857* repressor. Inversions tests were performed as described [2], for 20 min at 36°C or for 10 min at 37°C for strains grown in LB and for 10mn at 38°C for strains grown in SM. Briefly, overnight cultures were diluted 100-fold in appropriate media, and grown until OD600 0.3 and submitted to heat shock. A control sample was kept at 30°C. After 120 mn at 30°C cultures were plates on L medium containing ampicillin (50 μg/ml) and X-gal (80 μg/ml). Blue and white colonies were counted to estimated recombination efficiency.

### Transposition assay

Transposed strains were constructed as described in [20], by inserting *attL* and *attR* sequence in the same orientation in presence of a third *attB* site. Each site carried an antibiotic resistance gene in addition of the 5’ part of *lacZ* for *attL* and the 3’ part for *attR*. In the presence of the pTSA29-CXI strain were grown in LB at 30°C and shifted to 39°C for one hour to induce recombinase’s production. Cultures were plate on LB X-gal and blue colonies were selected. Transposition reactions were verified by PCR digestion and PFGE of NotI- digested genomic DNA.

### Fluorescence microscopy analysis

Strains were transformed by pFH2973 [29] carrying CFP-P1Δ30ParB and yGFP-pMT1Δ23ParB. Overnight cultures in LB were diluted 300 times in minimal medium A supplemented with 0.2% glucose and 0.12% casamino acid until they reached an OD 600 comprised between 0.05 and 0.1. Cells were then spotted on agarose pads and observed using a Zeiss axio observer Z1 microscope, an Evolve EM-CCD camera (Roper) and Axiovision software. Image analysis was performed using the MATLAB-based software MicrobeTracker Suite [30]: foci detection and position were characterized using spotfinder, and the interfocal distances estimated using Matlab.

## Supporting information

S1 Fig Identification of the interaction limit of the Ori MD. Graphical representation of the Ori MD (green box), NSR (gray line) and Right MD (red box). Coordinates of the *attR/L* sequences are indicated based on the zero pb reference of the MG1655 genome sequence. Recombination frequency between *att*L and *att*R obtained with an induction of 20’ at 36°C (light gray) or 10’ at 37°C (dark gray) are present on the histogram and correspond to an average of at least 3 independent experiments with their respective standard-deviation.
(EPS)

S2 Fig Localization of chromosomal loci in the Ori MD and NSR region depends on *oriC* position. MD maps of the *E*. *coli* chromosome are represented with the position of the Ori-4, and NSR-5 FROS-Tag in a WT (A), *Inv* (B), or *oriCZ* (C) configuration. For each panel, the position of foci is represented in function of the long axis of the cell (y-axis) and in function of the cell length (x-axis). Red dots and bars correspond to the localization of the NSR-5 loci and green to the Ori-4 loci. (D) Percentage of the population presenting different interfocal distances between the two different foci distributed from 0.2 to 0.5 μm.
(EPS)

S3 Fig Localization of chromosomal loci in the NSR region and Right MD depends on *oriC* position. MD maps of the *E*. *coli* chromosome are represented with the position of the NSR-1 and Right-2 FROS-Tag in a WT (A), or *oriCZ* (B) configuration. For each panel, the position of foci is represented in function of the long axis of the cell (y-axis) and in function of the cell length (x-axis). Red dots and bars correspond to the localization of the Right-2 loci and green to the NSR-1 loci. (C) Percentage of the population presenting an interfocal distance between the two different foci distributed among 0.2 to 0.5 μm.
(EPS)

S4 Fig Position of chromosomal loci in the Ori and Right MD depends on *oriC* position (cell with one focus). MD maps of the *E*. *coli* chromosome are represented with the position of the Ori-4, Right-2, FROS-Tag in a WT (top left panel), Inv (middle right panel), or *oriCZ* (bottom panel) configuration. For each panel, the position of foci in cell containing 1 focus (cell with 2 foci are show in Fig 5), are represented in function of the long axis of the cell (y-axis) and in function of the cell length (x-axis). Red dots and bars correspond to the localization of the Right-2 loci and green to the Ori-4 loci.
(EPS)

S5 Fig The genetic isolation between Right and Ori MD is not impacted by the presence of an ectopic *maoS* sequence in the NSR. Histograms of recombination frequency between *attL* and *attR* sequences in WT, oriCZ and maoS-NSR strains. The y-axis indicates the percentage of recombination between *attL* and *attR* sequences, obtained as described in Materials and Methods with 10’ induction at 38°C. * indicates that no *att*L*-att*R recombinant was obtained.
(EPS)

S1 Table The abbreviation cm, trim, rif, apr, kn refer to insertions conferring resistance to chloramphenicol, trimetoprime, rifampycin, apramycin and kanamycin. Frt refers to the FLP site specific recombination site. attR, attL, attB refers to the lambda site specific recombination site.
(DOCX)
