# Supplementary material for: Long range chromosome organization in Escherichia coli: The position of the replication origin defines the non-structured regions and the Right and Left macrodomains
Source: PLoS Genet. 2017 May 9;13(5):e1006758. doi: 10.1371/journal.pgen.1006758 (PMC5441646; doi:10.1371/journal.pgen.1006758)
Supplement: S1 Table — Frt refers to the FLP site specific recombination site. attR, attL, attB refers to the lambda site specific recombination site. (DOCX) [file pgen.1006758.s006.docx]

Table S1: Strains

| Strain | Relevant genotype/ position of *att* site | reference |
| --- | --- | --- |
| MG1655 | F- lambda- ilvG- rfb-50 rph-1 | Lab collection |
| MG1657 (FBG150) | MG1655 ΔlacZ ΔattB ::aadA | Valens 2004 |
| attL-R4 attR-R2 B^O-NSR^ | attLlC13(1099533) attR127(651775) attB(153248) | Thiel 2012 |
| RT | LC13-R127 B^O-NSR^ transposed *ΔattB’, ΔattR’, ΔattL’* | Thiel 2012 |
| attL-R1 attR-O4 | attL-R1 cm (-) 806549 attR-O4 kn (+)4371212 | This work |
| RT attL-R1 attR-O4 | RT attL-R1 cm (-) 806549 attR-O4 kn (+)4371212 | This work |
| attL-R1 attR-O5 | attL-R1 cm (-) 806549 attR-O5 kn (+)4476372 | This work |
| RT attL-R1 attR-O5 | RT attL-R1 cm (-) 806549 attR-O5 kn (+)4476372 | This work |
| \| attL-NSR2 attR-R1 \| \| --- \| | \| attL-NSR2 cm (-) 331520 attR-R1 kn (+)602656 \| \| --- \| | Valens 2004 |
| RT attL-NSR2 attR-R1 | RT attL-NSR2 cm (-) 331520 attR-R1 kn (+)602656 | This work |
| attL-NSR2 attR-R6 | attL-LC4 cm (-) 331520 attR 64 kn (+)1006773 | Valens 2004 |
| RT attL-NSR2 attR-R6 | RT attL-NSR2cm (-) 331520 attR-R6 kn (+)1006773 | This work |
| attL-NSR2 attR-T1 | attL-NSR2 cm (-) 331520 attR-T1 kn (+)1226270 |  |
| RT attL-NSR2 attR-T1 | RT attL*-NSR2* cm (-) 331520 attR-T1 kn (+)1226270 |  |
| attL-NSR2 attR-O3 | attL-LC4 cm (-) 331520 attR-R45 kn (+)4302200 | Valens 2004 |
| RT attL-NSR2 attR-O3 | RT attL-NSR2cm (-) 331520 attR 45kn (+)4302200 | This work |
| attR-NSR3 attR-O4 | attL9-1 tet (-) 362218 attR 32kn (+)4371212 | Valens 2004 |
| RT attR-NSR3 attR-O4 | RT attL- NSR3 tet (-) 362218 attR-O4 kn (+)4371212 | This work |
| attL-NSL2 attR-NSL1 B ^L-T2^ | attLR146 (-) 3697780 attR124 (-) 2892861 att B ^L-T2^ 1920110 | Thiel 2012 |
| LT | LR146-R124 B ^L-T2^ transposed *ΔattB’, ΔattR’, ΔattL’* | Thiel 2012 |
| attL-O2 attR-L2 | attL Trkd cm (+) 3928943 attR112 (-) kn 2470413 | Valens 2016 |
| LT attL -O2 attR-L2 | LT attL -O2 cm (+) 3928943 attR-L2 (-) kn 2470413 | This work |
| attL-NSL1 attR-O2 | attLC12 (-) cm 3250851 attR-O2(+) kn 4043280 | Valens 2004 |
| LT attL-NSL1 attR-O2 | LT attL-NSL1 (-) cm 3250851 attR-O2(+) kn 4043280 | This work |
| attL-NSL1 attR-NSL2 | attLC12 (-) cm 3250851 attR82(+) kn 3064833 | Valens 2004 |
| LT attL-NSL1 attR-NSL2 | LT attL-NSL1 (-) 3250851 attR-NSL2(+)3064833 | This work |
| attL-R1 attR-T2 | attL-R1cm (-) 806549 attR-T2 kn (+)1403134 | This work |
| RT attL-R1 attR-T2 | RT attL-R1cm (-) 806549 attR-T2 kn (+)1403134 | This work |
| RT attL-R1attR-T2 *ΔmatP* | RT attL-R1cm (-) 806549 attR-T2 kn (+)1403134 *ΔmatP::rif* | This work |
| attL-NSL1 attR-O2 | attL-NSL1 cm (-) 3250851 attR-O2 kn(+)4043280 | This work |
| LT attL-NSL1 attR-O2 | LT attL-NSL1 cm (-) 3250851 attR-O2 (+) kn 4043280 | This work |
| LT attL-NSL1 attR-O2 *ΔmatP* | LT attL-NSL1 cm (-) 3250851 attR-O2 (+) kn 4043280*ΔmatP::rif* | This work |
| attL-T2 attR-NSL2 | attL-T2 apr (-) 1914670 attR-NSL2 (+) kn 3064833 | This work |
| LT attL-T2 attR-NSL2 | LT attL-T2 apr (-) 1914670 attR-NSL2 (+) kn 3064833 | This work |
| LT attL-T2 attR-NSL2 *ΔmatP* | LT attL-T2 apr (-) 1914670 attR-NSL2 (+) kn 3064833 *ΔmatP::rif* | This work |
| attL-T2 attR-L1 | attL-T2 apr (-) 1914670 attR-L1 (+) kn 2050776 | This work |
| LT attL-T2 attR-L1 | LT attL-T2 apr (-) 1914670 attR-L1 (+) kn 2050776 | This work |
| LT attL-T2 attR-L1 *ΔmatP* | LT attL-T2 apr (-) 1914670 attR-L1 (+) kn 2050776 *ΔmatP::rif* | This work |
| attL-O3 attR-NSR3 | attL-O3 cm (-) 4024867 attR-NSR3 (+) kn 362295 | Valens 2004 |
| attL-O3 attR-NSR5 | attL-O3 cm (-) 4024867 attR-NSR5 (+) kn 570766 | Valens 2004 |
| attL-O3 attR-R1 | attL-O3 cm (-) 4024867 attR-R1 (+) kn 602656 | Valens 2004 |
| RT attL-O5 attR-NSR1 | RT attL-O5 cm (+) 4380221 attR-NSR1 (-) kn 130780 | This work |
| RT attL-O5 attR-R3 | RT attL-O5 cm (+) 4380221 attR-R3 (-) kn 678960 | This work |
| RT attL-R2attR-O7 | RT attL-R2apr (+)914197 attR-O7 (-) kn 4575276 | This work |
| RT attL-R3 attR-O7 | RT attL-R3 apr (+) 1002346 attR-O7 (-) kn 4575276 | This work |
| RT attL-O5 attR-R7 | RT attL-O5 cm (+) 4380221 attR-R7 (-) kn 130780 | This work |
| attR-R4 attL-NSR1 | attR17 (+) 806549 attL35 kn (-)160030 | Valens 2004 |
| ATTL-O1 attR-O8 | ATTL-O1(+) 3857785 AttR-O8 (-) 4638132 | This work |
| *InV* | ATTL-O1 AttR-O8 inverted ΔP’ ΔB’ | This work |
| *InV* attL -O2 attR-NSR3 | *InV* attL-O2 cm (-) 3928943 attR-NSR3 (+) kn 362295 | This work |
| *InV* attL-O5 attR-NSR3 | *InV* attL-O5 cm (-) 4380221 attR-NSR3 (+) kn 362295 | This work |
| attR-R4 attL-O6 | attR17 (+) 806549 attL28 kn (-) 4501855 | Valens 2004 |
| *InV* attL -O2 attR-R5 | *InV* attL-O2 cm (-) 3928943 attR-R5(+) kn 884387 | This work |
| *InV* attL-O5 attR-R1 | *InV* attL-O5 cm (-) 4380221 attR-R1 (+) kn 602656 | This work |
| *InV* attL -O2 attR-R6 | *InV* attL-O2 cm (-) 3928943 attR-R6 (+) kn 1006773 | This work |
|  |  |  |
| attL-O3 attR-NSL2 | attL-O3 cm (-) 4024867 attR-NSL2 (+) kn 3064833 | This work |
| *InV* attL-O5 attR-NSL5 | *InV* attL-O5 cm (-) 4380221 attR-NSL5 (+) kn 3286391 | This work |
| *InV* attL-O5 attR-NSL4 | *InV* attL-O5 cm (-) 4380221 attR-NSL4 (+) kn 3259579 | This work |
| attR17 attL-O7 | attR17 (+) 806549 attL11 kn (-)33910 | Valens 2004 |
| attL-R4 attL-T1 | attR17 (+) 806549 attL160 kn (-)1461878 | Valens 2004 |
| RCe504 | \|  \| *oriZ-cat-frt* \| \| --- \| --- \| | Ivanova 2015 |
| Ori Z attR-R4 attL-NSR1 | oriZ-cat-frt attR-R4 (+) 806549 attL-NSR1 kn (-)160030 | This work |
| OriZ attR-R4 attL-O6 | oriZ-cat-frt attR-R4 (+) 806549 attL-O6 kn (-) 4501855 | This work |
| OriZ attR-R4 attL-O7 | oriZ-cat-frt attR-R4 (+) 806549 attL-O7 kn (-)33910 | This work |
| OriZ attR-R4 attL160 | oriZ-cat-frt attR-R4 (+) 806549 attL160 kn (-)1461878 | This work |
| attR-R4 attL-NSR1 *ΔmatP* | attR-R4 (+) 806549 attL-NSR1 kn (-)160030 *ΔmatP::rif* | This work |
| attR-R4 attL-O7*ΔmatP* | attR-R4 (+) 806549 attL-O7kn (-)33910 *ΔmatP::rif* | This work |
| attR-R4 attL-O6 *ΔmatP* | attR-R4 (+) 806549 attL-O6 kn (-)4501855 *ΔmatP::rif* | This work |
| Ori-4 Right-2 | Ori-4 parS T1 cm (9883) Right-2 parS P1 trim (258144) | Espeli 2008 |
| *InV* Ori-4 Right-2 | *InV* Ori-4 parS T1 cm (9883) Right-2 parS P1 trim (258144) | This work |
| OriZ Ori-4 Right-2 | oriZ-frt Ori-4 parS T1 cm (9883) Right-2 parS P1 trim (258144) | This work |
| NSR-1 Right-2 | NSR-1 parS T1 cm (71279) Right-2 parS P1 trim (258144) | Espeli 2008 |
| OriZ NSR-1 Right-2 | oriZ-ftr-cat-frt NSR-1 parS T1 (71279) Right-2 parS P1 trim (258144) | This work |
| Ori-4 NSR-5 | Ori-4 pars T1 cm (9883) NSR-5 parsP1 trim (515143) | Espeli 2008 |
| *InV* Ori-4 NSR-5 | Ori-4 pars T1 cm (9883) NSR-5 parsP1 trim (515143) | This work |
| OriZ Ori-4 NSR-5 | oriZ-frt Ori-4 pars T1 cm (9883) NSR-5 parsP1 trim (515143) | This work |
